# Editorial Department

**Published:** 1854-01

**Authors:** 


Prof. Edward M. Moore, having completed his course upon Anatomy, at the Buffalo Medical College, is now in Columbus, Ohio, where he fills, temporarily, the chair of Surgery in the Starling Medical College, made vacant by the dangerous illness of Prof. Howard.

The sickness of Prof. Howard will be a subject of regret to those who know that gentleman, or who, like ourselves, have been in the habit of perusing his writings in the Ohio Medical Journal.

The class who have expected to listen to his teachings, will find his place most ably supplied by Prof. Moore, who carries with him the hearty good will, and sincere respect of the associates and students whom he has left in Buffalo. S. B. H.

